# COVID-19 Pandemic: Influence of Schools, Age Groups, and Virus Variants in Italy

**DOI:** 10.3390/v13071269

**Published:** 2021-06-29

**Authors:** Giovanni Sebastiani, Giorgio Palù

**Affiliations:** 1 Istituto per le Applicazioni del Calcolo Mauro Picone, Consiglio Nazionale delle Ricerche, Via dei Taurini 19, 00185 Rome, Italy; 2 Mathematics Department “Guido Castelnuovo”, Sapienza University of Rome, Piazzale Aldo Moro 5, 00185 Rome, Italy; 3 Department of Mathematics and Statistics, University of Tromsø, H. Hansens veg 18, 9019 Tromsø, Norway; 4 Department of Molecular Medicine, University of Padova, 35121 Padova, Italy; giorgio.palu@unipd.it

**Keywords:** COVID-19 pandemic, SARS-CoV-2 variants, school activities

## Abstract

The estimated smooth curve of the percentage of subjects positive to SARS-CoV-2 started decreasing in Italy at the beginning of January 2021, due to the government containment measures undertaken from Christmas until 7 January. Approximately two weeks after releasing the measures, the curve stopped to decrease and remained approximately constant for four weeks to increase again in the middle of February. This epidemic phase had a public health care impact since, from the beginning of the fourth week of February, the curve of the intensive care unit’s occupancy started to grow. This wave of infection was characterized by the presence of new virus variants, with a higher than 80% dominance of the so-called “English” variant, since 15 April. School activities in Italy started at different times from 7 January until 8 February, depending on every region’s decision. Our present data on the incidence of SARS-CoV-2 in different age groups in Italy are in agreement with literature reports showing that subjects older than 10 years are involved in virus transmission. More importantly, we provide evidence to support the hypothesis that also individuals of age 0–9 years can significantly contribute to the spread of SARS-CoV-2.

## 1. Introduction

There is enough literature data supporting the evidence that children are at low risk to be infected by SARS-CoV-2, as previously documented for SARS-CoV and MERS-CoV. Furthermore, when affected by COVID-19, children exhibit fewer symptoms, less severe disease, and extremely low fatality rate. Besides, children seem to transmit SARS-CoV-2 infection less frequently than adults [1,2,3,4,5,6]. These findings may largely reflect the presence of a young non-senescent immune system, actively stimulated by multiple vaccinations during childhood [7], and by the frequent natural exposure to seasonal common cold coronaviruses sharing cross-reactive epitopes with SARS-CoV-2 [8]. A number of reports have provided conflicting results about school opening and closure on amplifying or curtailing infection by SARS-CoV-2. Data vary according to students’ age, phase of the pandemic, region or country examined, and presence of other non-pharmacological interventions that might have played a role as confounding factors [9,10,11,12,13,14,15]. However, two recent studies in 131 and 41 countries showed that reopening of schools contributes to increasing the value of the reproductive number Rt up to 25%, while school closure contributes to decreasing it by 38% [16,17].

We recently [18] described how the exponential increase in the pandemic spread that was occurring in Italy in early October 2020, followed school reopening by 14 days, with a circulation of about nine million people. Noteworthy, two weeks is the average time between an individual infection and its registration as a positive case. No other event was coinciding, nor any additional suspension of mitigation measures was adopted within that time frame except schools. Our assumption was that school reopening impacted mainly on household transmission and was responsible for virus exponential spreading to the general population. Such an event would have then played a relevant role in pandemic amplification during the so-called “second wave”. In the present report, we describe the effect of school opening in Italy from January to February 2021, according to individual regions, after a prolonged closure for the Christmas holidays. Our data on SARS-CoV-2 incidence according to age and in the presence of the new English variant confirm our previous results on the relevant role played by the young students in the infection spread.

## 2. Materials and Methods

Analyzed data were publically available from official sources of the Istituto Superiore di Sanità and the Italian Civil Protection. Schools restarted in different Italian regions and autonomous provinces at various time periods, namely 7, 11, 18, and 25 January, and 1 and 8 February, 2021. Linear fitting of the percentage weekly increase of Intensive Care Unit (ICU) occupancy in the last weeks of February was performed in terms of the time when the school started pooling in four groups of data of the 21 Italian regions and autonomous provinces according to the time when school activity restarted. 

We also studied school data of the Piemonte region in the week from November 9 to 15, 2020, that were publically available in [19]. The incidence of SARS-CoV2 for that week was computed for all students and separately for students in the age group 11–19 years, school workers, and the general population. A hypothesis test about the independence of probability of being SARS-CoV-2 positive was performed by means of Chi-square statistics for pairs of groups. 

SARS-CoV-2 incidence curves were computed at the national level for the weeks from 11 January to 1 February 2021, and from 1 March to 18 April 2021, separately for the age groups 0–19, 20–29, 30–39, 40–49, 50–59, 60–69, 70–79, 80–89, and 90 or more years. We estimated the true value of the incidence by non-parametric linear regression [20]. Wilcoxon signed-rank test was performed to test the hypothesis about different curve patterns between groups.

## 3. Influence of School Activity on Health System

**Figure 1:**
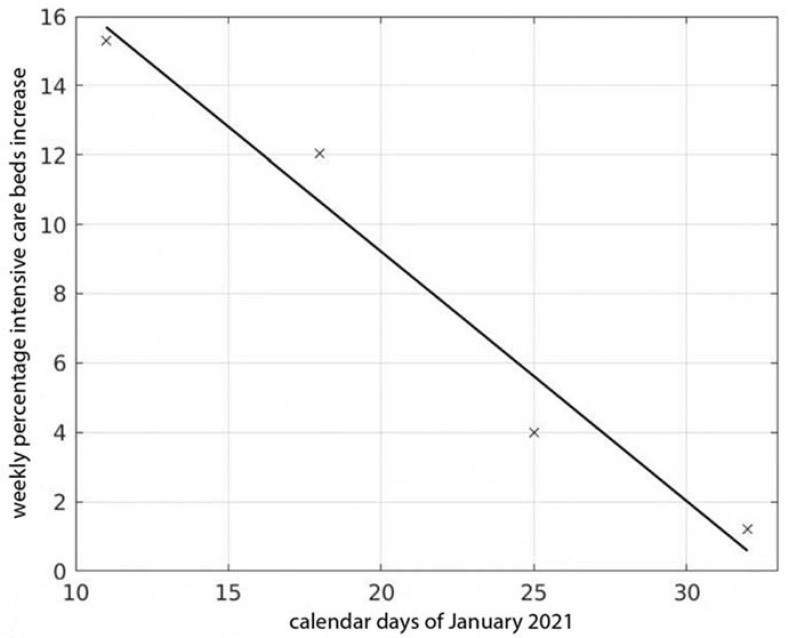
Percentage of weekly increase at the end of February 2021 in intensive care units’ occupancy for the cumulative curves of the four groups of Italian regions as a function of the respective calendar times when schools reopened in January.

We report here some results on the influence of school reopening during January and February 2021 on the COVID-19 epidemic in Italy. Bed occupancy in ICU was already adopted by us as a parameter that can suitably measure the pandemic intensity and the COVID-19 timeline of clinical evolution, a disease that, from the stage of early infection to the full-blown symptomatic phase needing ICU admission, can develop in approximately one month [21]. The result of the linear fitting of the percentage weekly increase of ICU occupancy in terms of the time when the school group started is shown in Figure 1. It is noticeable how the percentage weekly increase diminishes approximately linearly as the time of school start is prolonged.

According to our interpretation, this is additional evidence that school activities significantly contribute to the spread of SARS-CoV-2 [18]. Since rigorous measures were implemented in schools to limit virus spread, we think that this phenomenon is mainly due to transportation of students and school teachers and employees (about nine million people) under not sufficiently safe conditions and/or to other reasons, such as group gatherings outside schools for social activities. Once infected, students could have transmitted the virus to other members of their families (virtually eight million families were exposed), a condition that would have progressively increased the pressure on the health care system.

## 4. Age Groups and Epidemic Diffusion

When considering the results already published in the scientific literature, one can assume that subjects younger than 11 years are not involved in the process of SARS-CoV-2 diffusion [22,23]. A cue to this interpretation also comes from the analysis of school data of the Piemonte region that we performed here for comparison, considering the week of 9–15 November 2020. During such a time period, in fact, Piemonte reached the peak of SARS-CoV-2 positive subjects in the population after initial exponential growth and a phase with diminishing rate of increase due to reintroduction of containment measures. Data indeed show that students of age 11–19 years tested positive in a proportion of 42%, a statistically significant increase from the 35% value of the general population in the same week (*p* < 0.001). However, if subjects younger than 11 years are also included, we find that students were virus-positive in a proportion of 34%, a value that is not statistically different from that of the general population (*p* > 0.6). School workers and teachers were found positive in a proportion of 50%, and the difference from the general populations is, in this case, statistically significant (*p* < 0.001). The peak of incidence was reached fourteen days after reintroduction of remote teaching, a lag phase similar to the one that we had already seen when schools reopened in September, and the exponential phase took place [18]. It should be remarked that the individuals to be tested were not randomly selected so that a non-negligible fraction of the total positive subjects, i.e., those asymptomatic, was lost. These results show that, in the time interval considered, virus spread was higher for school workers, teachers, and students than in the general population. More specifically, for students, this was true when considering only subjects in the age group of 11–19 years.

**Figure 2:**
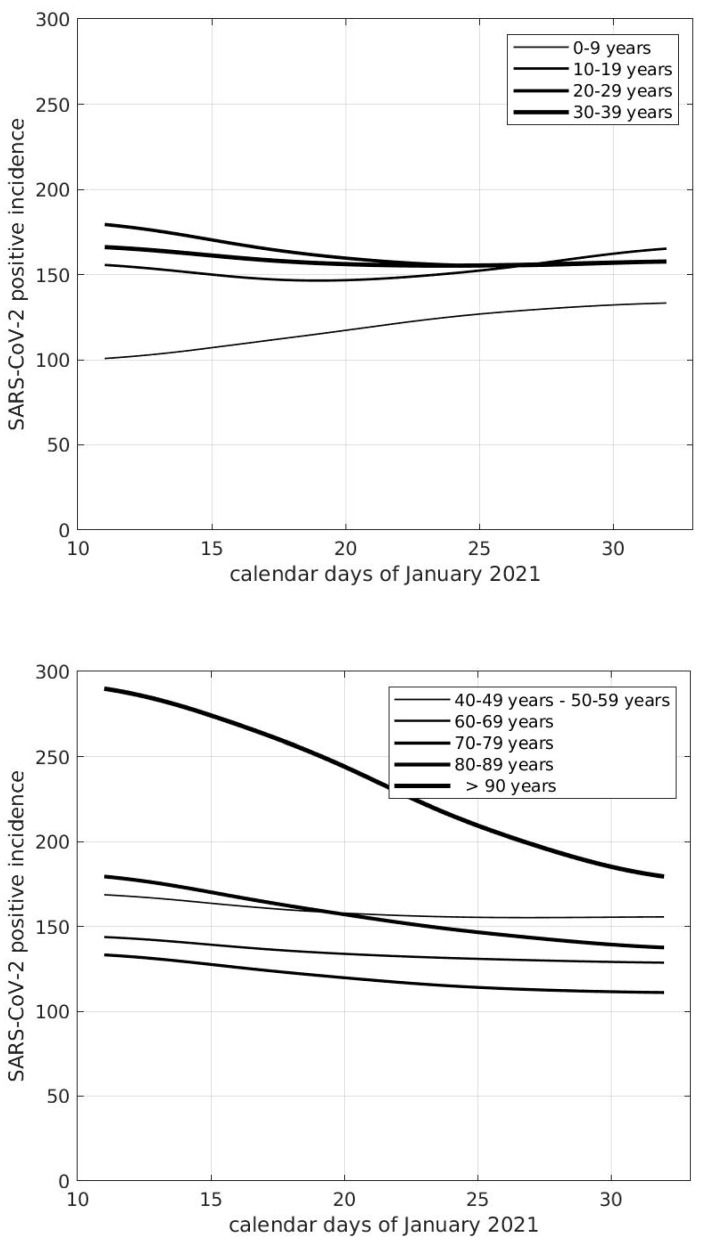
Curves of SARS-CoV-2 incidence in Italy referred to one week and 100,000 inhabitants of different age groups.

**Figure 3:**
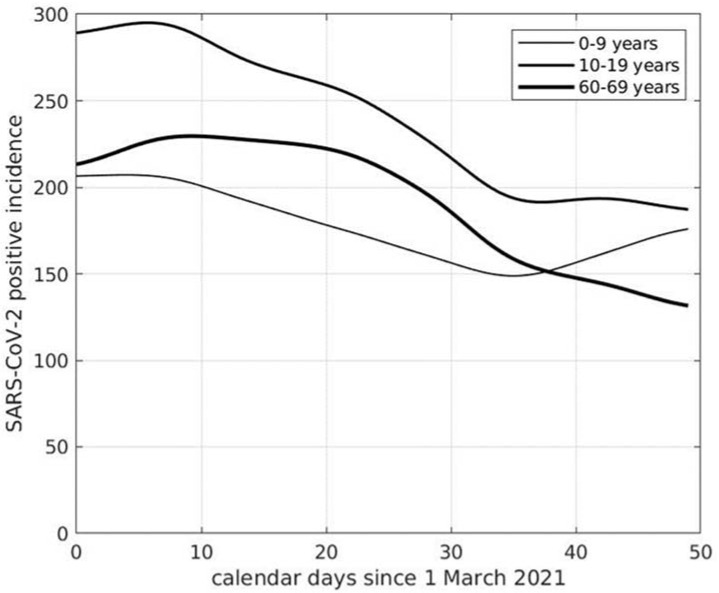
Curves of SARS-CoV-2 incidence in Italy referred to one week and 100,000 inhabitants of different age groups.

We now show results from the analysis of SARS-CoV-2 incidence data at the national level, providing evidence to support the hypothesis that also students younger than 11 years significantly contribute to the diffusion of the virus. In the top panel of Figure 2, we show the incidence curves of the age ranges 0–9, 10–19, 20–29, and 30–39 years. We notice that the curve for the range 0–9 years is increasing in the whole time-interval considered. The curve for the range 10–19 is the first to stop decreasing at about January 18. This happens roughly one week later for the curves of the ranges 20–29 and 30–39 years. The differences between the curve values in the first ten days of the time interval considered for the age group 0–9 years and the corresponding ones for each of the other three groups are statistically significant (*p* < 0.001). The curves for the ranges of people older than 39 years are shown in the bottom panel of Figure 2. As reported, ranges are decreasing in the whole time interval. It can be inferred from these results that the subjects first involved in the spread of the virus were those younger than 19 years, in particular, those younger than 10 years, mainly corresponding to students of primary schools. Similar findings are observed at the beginning of April 2021, as shown in Figure 3. The age range 0–9 years, in fact, was the only group showing increased SARS-CoV-2 incidence, a tendency that lasted up to the end of the month of April (not shown). In the second half of May 2021, COVID-19 cases showed a progressive and consistent decrease for any age category, a tendency that is still present in June at the level of every Italian region. This is probably due to the effects of restriction measures, the vaccination campaign, and the end of school activities. An additional contribution could be the increased social activities in the open air, where aerosol is absent, with consequent lower virus transmission probability.

## 5. Discussion

The data herewith presented partially contradict some results from a recent publication of Gandini et al. [24] that measured SARS-CoV-2 incidence in Italian students and employees, for the Italian regions and autonomous provinces, in the period from 12 September to 7 November 2020. In fact, compared to the general population, they found higher incidence among employees and lower incidence in students in the age range 6–13 years. Differently from our results of Piemonte, they found no incidence increase in students in the age range 14–18 years. In contrast with our analysis, they did not focus on the period where the incidence of infection was maximal. Moreover, during the first quarter of the time interval they considered, the incidence was not yet growing exponentially, but only linearly as during the weeks before. This is not an ideal choice if one aims to detect different growth curves between groups. In addition, Gandini et al. did not take into account that enhanced virus spread could be due to an overall increase in prevalence, notwithstanding how it was generated and in which population strata it was developed.

Our findings are also at variance with those that can be found in literature and that suggest that only students older than 11 years are involved in virus transmission [19,20]. Here, indeed, we show, with a survey conducted in two different time periods in the course of the pandemic, i.e., during the months of January and March 2021, that students of a much younger age can be involved in sustaining the pandemic wave in a significant manner. Such a finding could be explained by a higher exposure during fall and winter caused by school opening and by the circulation of new more infectious variants of the virus, in particular, the so-called "English" B1.1.7 variant. Its prevalence in Italy reached about 86% at the beginning of April starting from an 18% level at the beginning of February 2021 [25]. Such a variant has now become clearly the dominating lineage [26]. Being 30% to 90% more infectious and presenting new antigenic epitopes, this lineage could overcome natural defenses of young children represented by a lower number of receptors in the upper respiratory tract mucosa and the presence of cross-reactive anti-coronavirus antibodies [27]. Results similar to ours were obtained in a very recent serological survey conducted in Bavaria during the second wave of the pandemic [28]. The authors, in fact, showed that both pre-school and school children were particularly susceptible to SARS-CoV-2 infection, also largely due to the English variant, and that the cumulative frequency of infection was much higher than that reported in prior PCR-based virus surveillance. In conclusion, not only ours but also the Bavarian data on SARS-CoV-2 incidence in young pre-school and school children are discordant with previous literature data. An explanation for the above discrepancy may rest on the already mentioned facts that more infectious virus variants are now circulating and that asymptomatic cases are prevalent in childhood. Extensive studies with data from many different countries, as in [16,17], are needed to perform statistical inference on the hypothesis that subjects in the age range 0–9 years significantly transmit the English variant. Our report suggests that very careful control of emerging virus genotypes and containment measures should be considered to be put in place at the school level to contain virus spread and curb pandemic diffusion from schools to households and communities. This approach should be taken into account also in the perspective of other emerging variants that could become dominant, like the current one known as “Delta”.

## Data Availability

The sources of the measured data are indicated in the paper. The results of the analysis are available on request from corresponding author.

## References

[1] Snape M.D., Viner R.M. (2020). COVID-19 in children and young people. Science.

[2] Zimmermann P., Curtis N. (2020). Coronavirus infections in children including COVID-19: An overview of the epidemiology, clinical features, diagnosis, treatment and prevention options in children. Pediatr. Infect. Dis. J..

[3] Hoang A., Chorath K., Moreira A., Evans M., Burmeister-Morton F., Burmeister F., Naqvi R., Petershack M., Moreira A. (2020). COVID-19 in 7780 pediatric patients: A systematic review. EClinicalMedicine.

[4] Viner R.M., Mytton O.T., Bonell C. (2021). Susceptibility to SARS-CoV-2 infection among children and adolescents compared with adults: A systematic review and meta-analysis. JAMA Pediatr..

[5] Davies N.G., Klepac P., Liu Y., Prem K., Jit M., Pearson C.A.B., Quilty B.J., Kucharski A.J., Gibbs H., Clifford S. (2020). Age-Dependent Effects in the Transmission and Control of COVID-19 Epidemics. Nat. Med..

[6] Hippich M., Holthaus L., Assfalg R. (2021). A public health antibody screening indicates a 6-fold higher SARS-CoV-2 exposure rate than reported cases in children. Med.

[7] Fedeli U., Porreca A., Colicchia M., Schievano E., Artibani W., Biasio L.R., Palù G. (2021). Intravescical instillation of Calmette-Guérin bacillus and COVID-19 risk. Hum. Vaccines Immunother..

[8] Ng K.W., Faulkner N., Cornish G.H. (2020). Preexisting and de novo humoral immunity to SARS-CoV-2 in humans. Science.

[9] Forbes H., Morton C.E., Bacon S., McDonald H.I., Minassian C., Brown J.P., Rentsch C.T., Mathur R., Schultze A., DeVito N.J. (2021). Association between living with children and outcomes from covid-19: OpenSAFELY cohort study of 12 million adults in England. BMJ.

[10] Lee B., Hanley J.P., Nowak S., Bates J.H.T., Hébert-Dufresne L. (2020). Modeling the impact of school reopening on SARS-CoV-2 transmission using contact structure data from Shanghai. BMC Public Health.

[11] Viner R.M., Russell S.J., Croker H., Packer J., Ward J., Stansfield C., Mytton O., Bonell C., Booy R. (2020). School closure and management practices during coronavirus outbreaks including COVID-19: A rapid systematic review. Lancet Child Adolesc. Health.

[12] Park Y.J., Choe Y.J., Park O., Park S.Y., Kim Y.-M., Kim J., Kweon S., Woo Y., Gwack J., Kim S.S. (2020). Contact Tracing during Coronavirus Disease Outbreak, South Korea, 2020. Emerg. Infect. Dis..

[13] Heald-Sargent T., Muller W.J., Zheng X., Rippe J., Patel A.B., Kociolek L.K. (2020). Age-related differences in nasopharyngeal severe acute respiratory syndrome coronavirus 2 (SARS-CoV-2) levels in patients with mild to moderate coronavirus disease 2019 (COVID-19). JAMA Pediatr..

[14] Szablewski C.M., Chang K.T., Brown M.M. (2020). SARS-CoV-2 transmission and infection among attendees of an overnight camp-Georgia, June 2020. Morb. Mortal. Wkly. Rep..

[15] Stein-Zamir C., Abramson N., Shoob H. (2020). A large COVID-19 outbreak in a high school 10 days after schools’ reopening, Israel, May 2020. Eurosurveillance.

[16] Li Y., Campbell H., Kulkarni D., Harpur A., Nundy M., Wang X., Nair H. (2021). The temporal association of introducing and lifting non-pharmaceutical interventions with the time-varying reproduction number (R) of SARS-CoV-2: A modelling study across 131 countries. Lancet Infect. Dis..

[17] Brauner J.M., Mindermann S., Sharma M., Johnston D., Salvatier J., Gavenčiak T., Stephenson A.B., Leech G., Altman G., Mikulik V. (2021). Inferring the effectiveness of government interventions against COVID-19. Science.

[18] Sebastiani G., Palù G. (2020). COVID-19 and School Activities in Italy. Viruses.

[19] https://alessandroferrettiblog.files.wordpress.com/2020/12/dati-contagi-scuola.pdf.

[20] Eubanks R.L. (1999). Nonparametric Regression and Spline Smoothing.

[21] Olivieri A., Palù G., Sebastiani G. (2021). COVID-19 cumulative incidence, intensive care, and mortality in Italian regions compared to selected European countries. Int. J. Infect. Dis..

[22] LaRosa E., Djuric O., Cassinadri M., Cilloni S., Bisaccia E., Vicentini M., Venturelli F., Rossi P.G., Pezzotti P., Bedeschi E. (2020). Secondary transmission of COVID-19 in preschool and school settings in northern Italy after their reopening in September 2020: A population-based study. Eurosurveillance.

[23] Riley S., Ainslie K.E.C., Eales O., Walters C.E., Wang H. (2020). High prevalence of SARS-CoV-2 swab positivity and in-creasing R number in England during October 2020: REACT-1 round 6 interim report. medRxiv.

[24] Gandini S., Rainisio M., Iannuzzo M.L., Bellerba F., Cecconi F., Scorrano L. (2021). A cross-sectional and prospective cohort study of the role of schools in the SARS-CoV-2 second wave in Italy. Lancet Reg. Health Eur..

[25] (2021). Prevalenza Della Variante VOC2020/12/01, Lineage B.1.1.7 in Italia. Studio di Prevalenza 4-5 Febbraio 2021, Istituto Superiore di Sanità. https://www.epicentro.iss.it/coronavirus/sars-cov-2-sorveglianza-dati.

[26] Epidemia COVID-19, Aggiornamento Nazionale 31 Marzo 2021, Published 2 April 2021, Istituto Superiore di Sanità. https://www.epicentro.iss.it/coronavirus/sars-cov-2-sorveglianza-dati.

[27] Davies N.G., Abbott S., Barnard R.C., Jarvis C.I., Kucharski A.J., Munday J.D., Pearson C.A.B., Russell T.W., Tully D.C., Washburne A.D. (2021). Estimated transmissibility and impact of SARS-CoV-2 lineage B.1.1.7 in England. Science.

[28] Hippich M., Sifft P., Zapardiel-Gonzalo J., Böhmer M.M., Lampasona V., Bonifacio E., Ziegler A.-G. (2021). A public health antibody screening indicates a marked increase of SARS-CoV-2 exposure rate in children during the second wave. Med.

